# Morphometric study of acetabular depth and coverage and their clinical importance

**DOI:** 10.12669/pjms.38.8.5915

**Published:** 2022

**Authors:** Saneed Khaliq, Aisha Qamar, Samia Khalid Khokhar, Hammal Naseer

**Affiliations:** 1 Dr. Saneed Khaliq (M.Phil. Anatomy), Department of Anatomy, Mekran Medical College, Turbat, Balochistan Pakistan; 2 Dr. Aisha Qamar (M.Phil. Anatomy), Department of Anatomy, Bahria University Medical & Dental College, Karachi, Pakistan; 3 Dr. Samia Khalid Khokhar (M.Phil. Anatomy), Department of Anatomy, Bahria University Medical & Dental College, Karachi, Pakistan; 4 Dr. Hammal Naseer (Master’s in Public Health), Department of Community and Preventive Dentistry, Bolan University of Medical and Health Sciences, Quetta, Pakistan

**Keywords:** Acetabular depth, Central edge angle, Sharp angle, Hip dysplasia, Hip arthroplasty

## Abstract

**Objectives:**

To measure acetabular parameters and to compare parameters of acetabulum between men and women, and right and left sides.

**Methods:**

The study was conducted in PNS Shifa Hospital, Karachi from January to June 2021. Anteroposterior radiographs of both hips of 70 adults aged 20-70 years were included. The study consisted of 35 men and an equal number of women. We measured acetabular depth, femoral head coverage, acetabular inclination and acetabular depth and width. The acetabular depth was measured using Wiberg’s central edge (CE) angle and Sharp angle techniques. Femoral head coverage was determined using measurements between medial edge of hip joint space, lateral edge of acetabulum and femoral head. To determine acetabular inclination, Tönnis angle was measured. Acetabular depth & width ratio (ADR) was evaluated by dividing acetabular width by depth, and multiplying by 100.

**Results:**

There was significant increase in Sharp angle and Tonnis angle in females on the left side as compared to males. Wiberg’s central edge angle and acetabular width to depth ratio showed insignificant increase in males as compared to females, whereas values of femoral head coverage were insignificantly more in women.

**Conclusion:**

The acetabular parameters were insignificantly different in gender and between right and left sides, although mean values were within normal range. The larger ADR ratio in men was most likely due to increased body weight in them as compared to women.

## INTRODUCTION

The acetabulum is a cup shaped vault with a diameter of 52 mm.^1^ It is formed from the ilium, ischium and pubis parts of hip bone.^2^ The pubis contributes the anterior one-fifth, the ileum forms posterosuperior two-fifth and the ischium contributes to posteroinferior two-fifth of the acetabulum. It articulates with the head of the femur to form the hip joint. The acetabulum has a peripheral lunate articular surface and a central non-articular acetabular fossa on the floor.^3^ The articular fossa encircles all the articular surfaces except at the six O’clock position, where the circle is completed by the transverse acetabular ligament. The ligament forms the acetabular foramen. The acetabular fossa is filled with extra synovial fat pad and synovial membrane with a thickness of 2.0-4.4 mm.^4^

Different factors, like environment and ethnicity bring changes in morphology of acetabulum. The acetabular shape can be modified during intrauterine life due to interruption in its development or after birth due to harm to the cartilaginous lining of lunate articular surface. Dysplasia of acetabulum is the most frequent developmental anomaly of the hip bone, due to the maldevelopment of acetabulum, so that roof of acetabulum remains superficial, shallow and vertically oriented. This leads to reduced surface area for weight bearing, which thus bears increased force per unit area during walking and may result in early degeneration.^5^ Progressive acetabular changes have been used by the anatomists and forensic scientists to estimate age. With change in age, the fossa and lunate surface of acetabulum also vary. The accuracy for age detection was seen to be 89% in 10 years and 67% in five years interval changes. Parameters used for the age estimation were groove of acetabulum, rim shape and porosity, apex cavity, and acetabular fossa outer edge activity and porosity.^6^

The pelvic radiograph is the gold standard for initial assessment of patients with hip pain. Hip pathologies such as femoroacetabular impingement and hip dysplasia become symptomatic long before causing degenerative changes. In such cases hip radiographs are used for early detection. They also provide a guideline in hip preservation surgeries.^7^

Acetabular depth measurement has a vital role in determining acetabular pathologies^7^. Deep acetabula are more common in women (72%) than men (42%). Increased acetabular depth is associated with unstable slipped capital femoral epiphysis.^8^ Shallow acetabulum with insufficient femoral head coverage is associated with hip dysplasia. The dysplasia is caused by reduced contact between femoral head and acetabulum with resultant increase in joint load forces on the adjacent surfaces. This pressure leads to degeneration of joint and osteoarthritis.^9^ Radiographic measurements of acetabular depth are used as prognostic indicators in hip arthroscopy patients.^10^

There is scarcity of data regarding acetabular parameters in our population. In order to bridge this gap, this study was planned to measure different parameters of acetabulum as they are essential for hip joint replacement surgeries.

## METHODS

This cross-sectional study was conducted at PNS Shifa Hospital, Karachi from January to June 2021 after approval from Ethical Review Committee of Bahria University Medical and Dental College (BUMDC) (ERC-66/2021). Seventy adults of both genders 35 men and 35 women from 20-70 years with completely fused acetabula were enrolled in the study after written informed consent, using probability non-convenience sampling technique. Patients with hip dysplasia, osteoarthritis, rheumatoid arthritis, hip surgery, pregnancy and trauma were excluded from the study. Sample size was calculated using OpenEpi version 3.

The participants were recruited from non-orthopedic patients presenting to the radiology department for X-ray of pelvis and abdomen. Anteroposterior radiographs of both hips were used for measurements. The X-ray was done using Toshiba Rotanode™ system. All measurements were ascertained by the researcher. To eliminate bias all findings were confirmed by a consultant radiologist who was blinded to the study. Images were analyzed using Synapse digital image management system (Fujifilm Medical Systems, Tokyo, Japan). The observations were recorded in the evaluation proforma.

### Parameters:

**Fig.1a:**
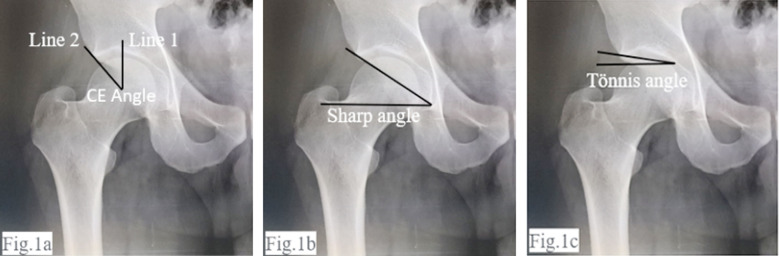
Wiberg’s central edge angle measurement. Fig.1b: Measurement of Sharp angle. Fig.1c: Measurement of Tonnis angle.

***1. Acetabular Depth:*** The acetabular depth was measured using Wiberg’s central-edge (CE) angle and Sharp angle/ lateral central edge angle (LCEA) techniques. The central-edge (CE) angle of Wiberg is a measure of depth of acetabulum and of the cover of formal head. Using Wiberg’s technique the center of the femoral head was established by drawing a circle around the inferior and medial margins. The central-edge angle was measured between two lines drawn from the center of the circle. The first line passed vertically along the longitudinal axis of the pelvis and the second line passed to the lateral acetabular rim (Fig.1a). Sharp developed a method for quantifying acetabular development known as the lateral central edge angle or Sharp angle. It was based on the theory that underdeveloped or pathological/dysplastic hips may concentrate increased force on the weight-bearing acetabular dome, predisposing this location to early degeneration of articular cartilage leading to premature development of osteoarthritis. An angle between 25° and 40° is taken as normal with 20°–25° being borderline. It is a radiographic measurement which shows coverage of superolateral femoral head by acetabulum. It is measured between a line drawn perpendicular to the transverse axis through center of head of femur and another line connecting the lateral edge of the acetabulum to the center of the femoral head^10^,^11^ (Fig.1b).

***2. Acetabular Inclination:*** To determine the acetabular inclination, the Tönnis angle was used. It was measured between a horizontal line from teardrop to teardrop and another line tangential to the sourcil (weight-bearing dome of the acetabulum)^12^ (Fig.1c).

***3. Femoral Head Coverage:***

**Fig.2a:**
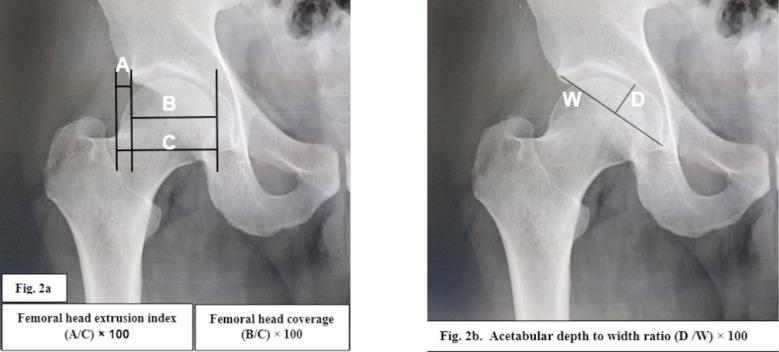
Measurement of head extrusion index and coverage. Fig.2b: Acetabular depth to width index.

The femoral head coverage (FHC) was measured using three vertical lines passing through the medial part of the hip joint space (line I), lateral edge of the acetabulum (line II) and lateral outline of femoral head (line III). The FHC was determined by the formula^13^: (Distance between lines I and II) ÷ (Distance between lines I and III) × 100 (Fig.2a).

***4. Acetabular Depth to Width Ratio:*** This ratio was calculated by determining acetabular width and depth. The acetabular width was determined between the inferior margin of its teardrop and its lateral margin. The acetabular depth was determined by a perpendicular line extending from the midpoint of the acetabular width to the center of the acetabular dome (Fig.2b). The acetabular depth to width ratio was calculated by the formula^14^: acetabular depth ÷ acetabular width × 100.

Data was analyzed using SPSS version 23. Independent-samples t-test was applied. *p*-value ≤ 0.05 was considered statistically significant difference.

## RESULTS

There were 70 participants in the current study, 35 males and an equal number of females. Thus 70 Anteroposterior pelvic radiographs were examined, and including both hip joints, a total of 140 hip joints were examined. The acetabular depth was measured using Wiberg’s central-edge angle and lateral center edge angle (LCEA) or Sharp angle. The mean value of Wiberg’s central edge angle (CEA) was 33.19° in the total study population, ranging from 21° to 44° in different subjects (Table-I). Comparison of right and left sides among the genders showed an insignificant increase on both sides in males as compared to females (Table-II).

**Table-I:** Comparison of acetabular parameters among study population (n=70).

Parameter	Mean Value	Upper limit	Lower limit
Wiberg’s central edge angle (degrees)	33.19	44.0	21.0
Sharp angle (Degrees)	37.11	46.5	27.5
Tönnis angle (Degrees)	4.46	14.5	.0
Acetabular Depth/Width Ratio (mm)	308.13	385.0	198.55
Femoral head coverage (mm)	83.94	92.67	76.31

P-value: significant ≤ 0.05*, Highly Significant ≤ 0.001**

Test applied: Independent-samples t-test.

**Table-II:** Comparison of Acetabular parameters among gender & between sides (n=70).

Parameter	Gender	Mean ± SD (Rt Side)	p-value	Mean ± SD (Lt Side)	p-value
Weber’s central edge angle (degrees)	Male	33.77±5.32	0.584	34.37±4.51	0.065
	Female	33.57±6.77		32.08±5.61	
Sharp angle (Degrees)	Male	36.20±4.02	0.196	36.20±3.50	0.006**
	Female	37.514±4.38		38.543±3.45	
Tönnis angle (Degrees)	Male	4±3.22	0.530	3.97±2.87	0.054
	Female	4.48±3.21		5.42±3.33	
Acetabular Depth/Width Ratio (mm)	Male	308.29±30.69	0.283	310.71±29.53	0.719
	Female	299.97±33.46		313.58±36.67	
Femoral head coverage (mm)	Male	83.47±3.57	0.121	83.43±2.99	0.626
	Female	84.86±3.84		83.53±3.81	

P-value Significant ≤ 0.05*,

** Highly Significant ≤ 0.001

Test applied: Independent-samples t-test.

This study showed that lateral center edge angle (LCEA) or Sharp angle had a mean value of 37.1° in the total study population (Table-I). There was highly significant increase (p=0.006) in LCEA in females (38.53°) as compared to the males (36.20°) on the left side. On the right side the increase was insignificant (37.51° in females and 36.20° in males) (Table-II).

Acetabular inclination was determined by measuring Tönnis angle. The mean Tonnis angle in the study participants was 4.46° (0° to 14.5° range) (Table-I). In males the angles were more on the right than left side (4°; 3.97°). In females the left side had more inclination than the right (4.48°; 5.42°). Among the two groups, females had higher acetabular inclination (Table-II).

Acetabular depth/width ratio (ADR) was 308.13mm in our study (Table-I). The left side was deeper than the right in both genders (males: 308mm Rt;310mm Lt; females: 299mm Rt; 313 Lt). The differences in the genders however were not significant (Table-II).

Femoral head coverage was 83.94 mm in the total study population (Table-I). Findings of 83.47mm on the right side and 83.43mm on the left side were observed in males. In females it was 84.86mm and 83.53mm on the right and left sides respectively. Variations in the genders were not significant (Table-II).

## DISCUSSION

Hip joint is not only indispensable to transmitting the weight of the human body to the femur, but also essential for movement. Structural anomalies in the acetabulum and its association with the head of femur can result in damage to the labrum as well as articular cartilage. This in turn can lead to degenerative diseases of the hip.^15^

Acetabular parameters are important for the stability of the hip joint. Studies have been carried out to determine these parameters to assist Orthopedic Surgeons and Biomedical Engineers in development of suitable hip joint prostheses.^16^ Also these measurements are different in different individuals and in various population groups.^17^

Abnormalities develop in biomechanics of hip due to the anomalies in acetabular parameters such as, depth, width and diameter resulting in dysplasia of hip, which in turn leads to instability of the hip joint, causing enhanced degeneration of the articular cartilage and ultimately ending in osteoarthritis. About 25.5% cases of osteoarthritis of hip joint result from acetabular dysplasia.^11^ Thus, measurements of acetabulum, such as depth of acetabulum, acetabular inclination and femoral head coverage are essential factors in restoration of hip mechanics back to normal in order to gain a good range of movement.^19^ So, the present study was designed to get a better insight of the acetabular parameters in our population.

In the present study, the mean value of central edge angle (CE angle) was 33.19°, with lowest and highest values being 21° and 44° respectively. In males, the mean value of CE angle was more on the left side as compared to the right, which was opposite in the females. This was probably because more people use their left leg for weight bearing irrespective whether they are right-handed or left handed.^19^ Concurrent results were found by Mohammad et al. when they examined pairs of hip bones and femur obtained from cadavers in the anatomy department of their institution.^20^ They also assumed that this was due to more weight bearing function performed by left hip joint during limb movements irrespective of limb dominance. However, the mean value of CE angle showed an insignificant decrease on the right side and insignificant increase on the left side in males as compared to the females. Another study done on Pakistani population showed results contradictory to our study as there was significant increase in central edge angle in males as compared to females (36.3±6.4° in males, 34.6±6.8° in females), whereas data of present study revealed equivocal values in males and females.^21^ Another study in which hip joints of 103 subjects (57 males and 46 females) were evaluated on CT scan of abdomen and pelvis done for various reasons other than hip related problems, showed similar results, with insignificant increase in CE angle among males as compared to females. This study showed a slightly less value of mean CE angle^14^ [31.1° (29.7°–32.4°)] as compared to our study (33.19°), most likely because it was done on Japanese population who have lesser height and weight as compared to our population.

Lateral center edge angle of Wiberg (LCEA) or Sharp angle is a method to assess morphology of acetabulum.^22^ Value less than 20° signifies dysplasia and more than 40° shows femoroacetabular impingement. In the present study, the mean value of Sharp angle was 37.11°, although it ranged from 27.5° to 46.5°. Results of this study are in accordance with another study^15^ who found a mean value to LCEA to be 35.9° although cases ranged from 20° and 50°.

When compared among gender, our results found an increase in Sharp angle among females as compared to males, which was highly significant on the left side and insignificant on the right side. Another study investigated the Sharp angle in 91 cases of hip pain, younger than 60 years of age, including 61 women and 30 men by performing MRI. Among these, mean value of sharp angle was 35.9^°^ in women, which was slightly lower as compared to our study, probably because these cases were suffering from different diseases as compared to ours which mainly comprised of healthy controls, whereas value was 36.4° in men which was same as in our study. This study also showed higher values of sharp angle among women^10^ as compared to men, analogous to our study.

The acetabular inclination is used to evaluate the extent of femoral head coverage by the acetabulum. Roof or dome of acetabulum is the part which bears weight and anatomic reconstruction of the acetabular dome with reduction of head of femur is the eventual objective of operative as well as nonoperative treatments.^24^ Upper limit of Tonnis angle is 10°. A Tönnis angle more than 10° shows dysplasia of the acetabulum.^25^ In the present study, the mean acetabular inclination was 4.46°, although it ranged from 0° to 14.5°. Similar results were determined by Zacharia & Fawas (2017)^12^ who observed a mean acetabular inclination of 4.1° in 800 AP pelvic radiographs in a tertiary care hospital of patients aged between 20-60 years, although angle varied from 1° to 9°. The inclination is inversely proportional to the femoral head coverage, higher the Tonnis angle, lesser is the coverage for the head of femur as in developmental dysplasia of hip. Decreased angle manifests over coverage of the head of femur and leads to femoroacetabular impingement.^15^

Our results also showed significant increase in Tonnis angle on the left side in females as compared to males whereas insignificant increase in Tonnis angle in females on the right side was observed, and the mean ranged from 3.97° to 5.42°. Another study^10^ demonstrated much higher values of Tonnis angle, conflicting to our results, which were 8±1.45°in women and 8.6±0.32° in men. The value of Tonnis angle was insignificantly higher in men in comparison to women, which was also in contradiction to our results.

Our study showed the mean value of femoral head coverage (FHC) as 83.94mm, although it ranged from 76.31 to 92.67. Results also showed an insignificant increase in FHC on the right side, whereas an insignificant decrease on the left side in females when compared with males. Mimura et al (2017)^14^ showed a slightly lower value (81.9), although they also had insignificant increase in value of femoral head coverage in females as compared to males in their CT scans of pelvis.

Our study showed insignificant increase in the acetabular depth to width ratio among males as compared to females with a mean value of 308.13mm (385.0-198.55). This was most likely because males have a significantly increased height as compared to the females, therefore this difference was also revealed in the acetabular dimensions among them.^24^ Mimura et al^14^ demonstrated similar findings in CT scans of hip joints, with almost the same value of mean acetabular depth to width ratio. However, their study revealed an insignificant increase in this value in females as compared to the males. All the values were within normal ranges, as they included normal subjects who did not suffer from hip pain or any other problem related to hip joint. A value of acetabular depth to width ratio less than 250 is most likely a representation of dysplasia of hip.^8^ The value of acetabular depth to width ratio had almost similar values on the right and left side in the current study. Similar findings were observed by another study who found that values of acetabular depth were close to each other in sacra of both sides.^13^

### Limitations:

This was a single center study. The sample size was small because of time constraint in the study.

## CONCLUSION

The observations of the present study have shown that acetabular parameters vary in males and females and between the right and left sides. The value of acetabular depth to width ratio and Wiberg’s central edge angle was more in men as compared to women, whereas values of Sharp angle, acetabular inclination and femoral head coverage were insignificantly higher in females than males. The mean values of these parameters were within normal range as these measurements were obtained from healthy individuals, in which all hip pathologies were excluded. The prostheses used in our population are based on measurements of the bones in western population which differ from those of ours. Thus, these measurements can form a base line for the Biomedical engineers to construct well fitted prostheses.

### Author’s contribution:

**SK:** Conceived, designed, prepared manuscript. Responsible and accountable for the accuracy and integrity of the study.

**AQ:** Write up, editing and final approval of manuscript.

**SKK:** Involved in manuscript preparation.

**HN** Statistical data analysis and write up of manuscript.
